# “I am going to break this logic of fear!”: Activism and subversive care at the periphery of Fortaleza, Brazil

**DOI:** 10.1111/jlca.12676

**Published:** 2023-05-29

**Authors:** Luminiţa‐Anda Mandache

**Affiliations:** ^1^ Senior Lise Meitner Fellow, Center for Ethics and Poverty Research University of Salzburg Salzburg Austria

**Keywords:** women, activism, periphery, violence, care, mulheres, ativismo, periferia, violencia, cuidado

## Abstract

Based on ethnographic work conducted between 2015 and 2022 at the periphery of Fortaleza, in Northeast Brazil, this article analyzes the work of community activists as a form of subversive care. Women activists, many of whom work for the local public clinics, as social workers with local NGOs, or as schoolteachers, challenge dominant narratives presented in the media and political discourses about their neighborhood as being poor and therefore violent. By establishing relationships of mutual trust with gang members and humanizing them, women activists “challenge the logic of fear” and maintain presence in areas controlled by the gangs to direct the economically vulnerable toward existing public resources. Activists’ understanding of urban violence is informed by participation in collective action and living together with gang members and their families. These experiences lead activists to see urban violence as the symptom of systemic inequalities that require systemic changes.

## Translated Abstract

A partir de uma pesquisa etnográfica realizada entre 2015 e 2022 na periferia de Fortaleza, no Nordeste do Brasil, este artigo analisa o trabalho dos ativistas comunitários como cuidado subversivo. Mulheres ativistas, muitas das quais trabalham como agentes de saude, assistentes sociais em ONGs locais ou professoras, desafiam as narrativas dominantes apresentadas na mídia e os discursos políticos sobre seu bairro como sendo pobre e, portanto, violento. Ao estabelecer relações de confiança mútua com os membros das gangues e humanizá‐los, as mulheres ativistas “desafiam a lógica do medo” e mantêm presença em áreas controladas pelas gangues para direcionar moradores economicamente vulneráveis aos recursos públicos existentes. O entendimendo dos ativistas da violência urbana é resultado da participação em ações coletivas e da convivência com membros de gangues e suas famílias. Essas experiências levam os ativistas a ver a violência urbana como sintoma de desigualdades sistêmicas que exigem mudanças sistêmicas.

On a quiet morning in September 2017, I found myself having a *cafezinho* (sweet coffee) in Beatriz's home with Lucia, one of her closest friends. Both Beatriz and Lucia were active members in the liberation theology movement at the periphery of Fortaleza, Brazil, in the 1980s. Beatriz continued her work as a popular educator^1^ working for different nongovernmental organizations (NGOs) in the city. Lucia^2^ retired not long ago from almost 30 years of work as a schoolteacher in Conjunto Palmeiras, a neighborhood on the periphery of Fortaleza. As chickens and cats roamed among our legs, in the small kitchen with dirt floor and a rooftop from which rays of sun would reach the table, they were complaining about today's residents from Conjunto Palmeiras.

In their eyes, the residents and even some leaders seem paralyzed by the fear of drug‐related violence. Their complaint was that residents act like violence is all “there is” and “can't see beyond that.” Instead of going along with the fear, Beatriz takes the bus regularly from her home to reach the Association of Women, where she talks with women, many of whom have lost someone to the *mundo do tráfico* (the world of drug trafficking), about their daily problems. Lucia, even though retired, spends a lot of her time visiting her schoolteacher friends, her *companheros de luta* (activist friends), and attends the meetings of a few local NGOs. They remember that in the 1980s, soon after the neighborhood was created, residents would organize by blocks, meet weekly, support each other, and discuss ways to solve their problems. Today people are reluctant to talk about local problems, including the insecurity that accompanies residents every day. Lucia and Beatriz blame this lack of civic engagement on a paralyzing fear that prevents most people from leaving their homes. This fear comes from consuming media that presents their neighborhood as a “hyperfavela,” or as an impoverished place where violence is all there is at the margins of the city (Robb Larkins, 2015). Instead, they continue their work in places where few of their community members dare to go, and they challenge narratives promoted by the media describing their neighborhood as the poorest and one of the most violent in the city.

While most studies on urban violence focus on the cities of Rio de Janeiro or São Paulo, less is known about how urban violence permeates social life in Northeast Brazil, historically one of the poorest regions of the country, and how residents respond to this unprecedented moment that disrupts their communities. Homicide rates in Brazil are among the highest in the world and registered a 24 percent increase between 2007 and 2017, and a 159 percent increase during the same period in the northeastern state of Ceará. The number of violent deaths tripled in Fortaleza, the capital city of Ceará, in great part due to the expansion of drug gangs originally situated in the southern part of the country (Madeiro and Costa, 2017).

In this article, I discuss how activists in Conjunto Palmeiras respond to, make sense of, and continue their work in this context of violence that reconfigures their communities. I focus on activists’ understanding of this new surge in violence, an understanding that shapes how they choose to relate to the drug gangs. I use the term *subversive care* to talk about activists’ practices that are gendered, politicized, and are manifested as care for their communities and especially so for those labeled by society as *marginais* (marginals or criminals). These practices are subversive because they challenge narratives about peripheries as inherently violent and dangerous. To destigmatize the urban periphery, activists reach the most vulnerable members of their communities and spend time guiding families in areas underserved by public institutions or the third sector. In this work, they rely on bonds of mutual trust with community members who recognize their work as care. In so doing, they create new relationships with, and narratives about, their communities and stigmatized social groups.

To investigate these activist practices, I conducted 17 months of ethnographic fieldwork in Conjunto Palmeiras, between 2015 and 2022, and I collected over 60 interviews with local activists and residents from different parts of the neighborhood. Part of my ethnographic work included teaching English classes to schoolteachers and low‐income teenagers and women and joining health agents in their daily visits. I spent 2015 conducting ethnographic fieldwork in Fortaleza; shorter research trips in 2016, 2017, and 2022 allowed me to follow closely the work of local activists and refine my understanding of how activist practices intertwine with personal life experiences. When not in Brazil, I communicated regularly with research participants through social media. In this article, I first situate subversive care in anthropological literature about Latin American women's agency. Then I introduce the context of this study, emphasizing the tension between ongoing poverty and activism in the region. Finally, I discuss the life trajectories of community activists at the margins of Fortaleza and illustrate ethnographically the origins and expressions of subversive care.

## MATTERS OF SUBVERSIVE CARE: ACTIVISM AND POLITICAL SUBJECTIVITIES

Latin American women's agency has been a constant topic of analysis and discussion in social sciences and popular literature; this is due in great part to the region's tension between ongoing women's activism and persistent gender inequality. Sociological studies on how Latin American women engage in reshaping the national political agenda at historical moments, such as during dictatorship regimes of the twentieth century or the transition period, are numerous. Such studies focus, for example, on women's movements articulated around class, gender, and race (Alvarez, 1990; Burdick, 1998; Thayer, 2010), on women's participation in the liberation theology movement and Christian Base Communities (Drogus, 1990, 1999; Drogus and Stewart‐Gambino, 2005; Hewitt, 2000), or on urban struggles to improve low‐income communities’ access to public resources during democracy (Auyero, 2003; Junge, 2018; Perry, 2013).

To understand the work of women at the margins of Fortaleza, I considered the theoretical concepts of activism, or “militancy,” and “resistance.” My focus is not identities or subjectivities but rather how they shape activist practices in a moment in Northeast Brazil marked by unprecedented levels of homicide rates. The term *resistance*, the emblem‐concept in literature on social movements (Ortner, 2016) is, I believe, unable to capture the nuance of the practices that I describe. Speaking of contemporary African states, James Ferguson (2007) remarks that a vertical topography of power situating the state “up there” and the community “on the ground” with civil society “in the middle” naturalizes the state's authenticity while also loading with nostalgia the “grassroots” or “the local.” Instead, Ferguson suggests acknowledging “that new forms of transnational connections increasingly enable ‘local’ actors to challenge the state's well‐established claims to encompassment and vertical superiority in unexpected ways” (397). All the women discussed here gain their knowledge about feminism, politics, and activism from social media groups that are transnational in nature. NGOs in their neighborhood are also embedded in larger transnational networks that circulate ideas and shape local political imaginaries.

A second critique of the concept of resistance, also building on Ferguson's comments, targets what we mean by “state.” What does resistance mean in a state led by a progressive president supported by social movements? Or, in the words of a senior community activist in Conjunto Palmeiras in 2015, “Who are we going to resist now? We are the state.” The activist was referring to the Leftist governance of the Workers’ Party between 2003 and 2016 and to the situation when the party that he and the movements he was part of supported left them disappointed. Under pressure from the opposition parties, corruption cases, and the decline of the economy, joining protests against the Workers’ Party would have translated into support for the opposition. Similarly, speaking about social movements in Brazil, James Holston (2008) rejects the state vs. social movements dichotomy, emphasizing the entanglement rather than the tension between the two.

Sian Lazar's (2017) term *militancia* (militancy) captures an understanding of political subjectivities together with the values and dispositions that turn such citizens into actors of change and emphasizes the social affective ties that frame militant work. Yet not all activists voiced here see themselves as militants. Their participation in collective action is erratic and fragmented; many left the organizations that inspired their work today years ago. Many are not affiliated with any movement or political party but join social movements episodically. Social scientists have employed the terms *resistance* and *militancy* to refer to participation in organized social movements or entities that opposed politics that work to the detriment of social justice. The political practices I discuss here are unlike it, in that they are subtle, detached from consistent participation in organized, collective action, and subversive.

Last, none of these concepts account for the influence of different forms of spirituality or religious institutions on the creation of activist voices. For example, the liberation theology movement is often considered either limited to the twentieth century or “failed.” Yet anthropological scholarship on its persistence and legacy shows the contrary. Even though in the mid‐1990s Pope John Paul II declared the end of liberation theology, today progressive Catholics remain important institution builders in Brazilian social life and continue activist work in the area of antiracism, women's rights, access to land, and access to healthcare (Burdick, 2004). Additionally, Jan Hoffman French's (2006) study in the rural areas in Northeast Brazil illustrates the importance of the liberation theology doctrine in defining the church's local relation to the poor. In my own ethnographic work in Fortaleza, I encountered liberation theology priests who still mobilize poor communities such as Bom Jardim (Greenfield, 2010) and Serviluz. In Conjunto Palmeiras, the last liberation theology priest left around 2000, and mobilization around the Catholic church declined mostly after this date. These facts indicate that, contrary to popular belief, liberation theology continues to exert influence on grassroots activism in Brazil. Here I do not see the liberation theology movement as limited to a set of institutions or key actors (e.g., the clergy) but as an ideology about change and justice in Brazil that persisted over time through the work of individuals inspired by this movement.

I suggest a concept of care that speaks about activist practices that are affective, gendered, and politicized. Their affective and gendered aspects relate to values that shape women's identities, many of which can hardly be disentangled from Catholicism and are deeply embedded in Brazilian society. Women are perceived as caregivers of their families, and when in the role of social workers or activists, also as caregivers of their communities. In their own words, women *cuidam* (care) for their neighborhood. Here I draw on Joan Tronto's definition of care as a political concept, as “a species activity that includes everything that we do to maintain, continue and repair, our ‘world’ so that we can live in it as well as possible” (1996, 142). This definition sits on the understanding that care is not limited to private life but is actually a very present public element and therefore contains the potential to broaden existing definitions of the political.

Women's care is also politicized, and it differs from charity or other religion‐based forms of caring. Subversive care is politicized because it is informed by participation in different movements, such as the local liberation theology movement around Christian Base Communities, the local struggles for urbanization in the 1980s and 1990s, the regional feminist movement, the Black movement, the solidarity economy movement, or Left‐wing political parties. As I show later in this article, their care is informed by a rights‐driven approach to justice and change.

More importantly, care is subversive, understood as the opposite of obedient and compliant, and important for its symbolic value to challenge assumptions about the urban periphery. Subversive care, being culturally and historically embedded, cannot be regarded as separate from the process of auto‐construction of Brazilian urban peripheries and insurgent citizenship developed in the twentieth century (Holston, 2008). Holston argues that this process, both material and symbolic, turned peripheries into spaces of alternative futures and ended up transforming national democratization. We could argue that subversive care emerges as one extension of insurgent citizenship, to serve other purposes, in the twenty‐first century. Just like insurgent citizenship, subversive care illustrates that urban peripheries are not just physical and geographical spaces but also arenas of sociability and cultural expression, and the sites of political projects of emancipation (Kopper and Richmond, 2020). This happens in a context where mass media and state discourse and policies equate urban violence with marginality and moral degradation. Processes of “othering” are part of these narratives, as the popular Brazilian saying “*Bandido bom e bandido morto*” (a good bandit is a dead bandit) suggests. Outcomes of these practices and discourses can be measured in the high numbers of Black and Brown teenagers from the margins of large cities killed every day by the Brazilian police and parastatal actors. It is in this climate that local activists choose to humanize teenagers who join drug gangs. Activists’ understanding of teenagers is informed by an intimate understanding of vulnerable households and by their political consciousness, developed through participation in different social movements. In the analysis that follows I focus on understanding how activists gain mutual respectful relationships with the gangs and “challenge the logic of fear” to be present in vulnerable areas of their communities.

## NEW PATHS OF THE DRUG TRADE AND URBAN FRACTURES

Although international media reports and popular culture promote Rio de Janeiro, Salvador de Bahia, or São Paulo as exotic tourism destinations, in the past years, a new city appeared in reports and news about Brazil: Fortaleza. The investment in tourism since the 1980s (Belmino, 2018) opened the city of Fortaleza up to a global flow of tourists and a new set of problems (Gondim and Hallewell, 2004). Sílvia Helena Belmino's (2018) study on the transformation of Fortaleza from a place of misery and capital of drought into an exotic tourist destination describes this strategy as part of a larger political and marketing project initiated under Brazil's last years of dictatorship. This project of change sat on a long history of systemic inequalities; therefore, Fortaleza's current uneven urban development is directly related to social inequality in the countryside, particularly nonlandowning agriculturalists who face persistent drought, under‐industrialization, and low levels of capital (Garmany, 2011).

It is not surprising then that in 2010 extreme inequality in Fortaleza was among the highest in the world with a Gini coefficient of 0.61 (López Moreno et al., 2010). The intensification of the drug trade in the area, coupled with inequality, contributed to increased homicide rates after 2006. In 2015 Fortaleza registered a homicide rate of 46.5 deaths for 100,000 inhabitants, but the number decreased by 14.2 percent the following year, thanks to a temporary peace agreement between the drug gangs, only to increase to 86.7 deaths per 100,000 in 2018 according to data from the Brazilian Forum for Public Safety (Cerqueira et al., 2019). According to a United Nations Children's Fund (UNICEF) study, in 2014 Fortaleza had the highest number of teen homicides in Brazil; 97.95 percent of the victims were boys, more than 65 percent identified as Black or *pardo* 3 (Brown) and lived at the periphery of the city (Rodrigues Aguiar and de Holanda Altamirano, 2017). These figures illustrate once more the tight connection between race, class, and urban violence in Brazil. The arrival of the large gangs from other regions of Brazil, such as Comando Vermelho or Primeiro Comando da Capital (Paiva, 2019), coincided with a sharp decrease in Workers’ Party popularity, numerous corruption scandals, Brazil's 2014 economic crisis, and the impeachment of then President Dilma Rousseff. At the community level this arrival was experienced as constant fear, forced dislocation, lack of freedom of movement between neighborhoods or between different regions of the same neighborhood, and restricted access to public infrastructure (Oliveira Silva Filho and Monteiro Mariano, 2020).^4^

Conjunto Palmeiras is a neighborhood created in 1973 by gentrification in the coastal area of Fortaleza. Its first residents remember how in the 1970s trucks brought around 3000 people with all their belongings from different parts of the city: some from the coastal area, some from the flooded parts of the city, and others from places occupied today by shopping malls or hotels. They were dumped in a place that had no water, electricity, or roads that could connect it to the rest of the city. Over the span of 40 years, the population in Conjunto Palmeiras has increased tenfold. Today's Conjunto Palmeiras has a center with a main square, shopping areas, and, as many residents will tell you, “even a bank,” referring to the Palmas Bank, a solidarity economy project that gained an international reputation for implementing a local currency. The solidarity economy project builds on a history of collective action that residents in Conjunto Palmeiras are very proud of. Among administrators working in Fortaleza's public administration, Conjunto Palmeiras is known as an example of grassroots organizing, since its leaders regularly attend public meetings or partake in participatory planning projects, and the place has been the object of numerous studies on urban “resistance” (Paulino, 2019). The neighborhood is particularly known for efforts made between the 1980s and 2000s by the Association of Residents to gain access to public infrastructure through protests and alliances with other grassroots organizations at the periphery of the city.

During its almost 50 years of existence, Conjunto Palmeiras has been reshaped by economic, demographic, and political changes. Using data from the 2010 census, the Municipality of Fortaleza measured the human development index of its 119 neighborhoods and concluded that Conjunto Palmeiras had the lowest scores on education, income, and life expectancy (de Castro, 2014). This news was heavily promoted by the local media with maps of the city marking in red the periphery of Fortaleza, where Conjunto Palmeiras is situated. When drug‐related violence increased in the city, popular television and local newspapers again promoted new maps with headlines announcing that “Painful homicides are concentrated in the poor and peripheral neighborhoods of Fortaleza” (Diārio do Nordeste, 2013). Such representations of Conjunto Palmeiras, and the periphery of Fortaleza in general, were met by residents with attitudes ranging from distrust to anger, and they often tried to convince me, a visitor, of the invalidity of these studies. In exchange, many residents talked about neighborhoods that were as “dangerous” as theirs but did not appear in the ranking or argued that “violence exists everywhere.” Their comments spoke about the role of the media in reproducing and reinforcing socio‐spatial binaries that illustrate structural inequalities.

## THE CRAFTING OF SUBVERSIVE CARE THROUGHOUT THE LIFE COURSE

Most research on the impacts of drug‐related violence on civic action focuses on southern Brazilian cities and argues that drug‐related violence and its counterpart, fear, represent challenges to grassroots democratic civic action. To illustrate this argument, various scholars point to practices such as the appointment to local leadership positions of persons supported by drug gangs (Leeds, 1996), to the financing of public work projects by drug gangs, or their close relationship with community organizations (Arias, 2006; Gay, 1994)—practices that in the end fuel discontent and skepticism toward community leaders (Savell, 2015). Others argue that drug‐related violence makes local leaders reticent to meet and work (Zaluar and Ribeiro, 1995). Here I describe how particular life experiences in the neighborhood shape political subjectivities that lead to subversive care toward residents considered “marginals.” The moral universe of the city, constructed by actors and practices including political parties, public policies, or the media, distinguishes between *marginais* and *cidadãos de bem* (good citizens). This distinction played a major role in former President Jair Bolsonaro's political discourse and became the object of public debate and commentary in Brazil in the past years (Medeiros et al., 2021). Such distinctions cannot be seen separated from center–periphery inequalities that characterize all Brazilian cities, with poor residents, often at the margins of the city, and the middle‐ and upper‐class residents occupying the economic and political centers. Urban peripheries are presented as the source of urban violence that needs to be “pacified” through the work of the police. Subversive care is crafted as an alternative morality, which dictates that the *marginais* are worthy of respect and dignity. To illustrate how such morality takes shape in relation to particular life trajectories, I describe the life trajectory of one activist.

In 2017, Raimunda, a woman in her 50s, lived in Conjunto Palmeiras, where she was employed as a health worker. She moved to Fortaleza as a child with her mother from the interior of the state to seek a better life, part of a larger movement of internal migration that led to the increase of Latin American cities toward the middle of the twentieth century. In her youth, she worked for a state‐funded institution offering support to women victims of domestic violence. Through this experience she became part of the women's movement and the local liberation‐theology‐inspired movement, both important during Brazil's redemocratization process (Alvarez et al., 1998). In 2003, through an *invazão* (collective occupation of land), Raimunda moved to Conjunto Palmeiras. At that time, the community—today a feared part of the neighborhood assumed to be the home of gang leaders—lacked access to water, electricity, streets, schools, and healthcare, and much of this infrastructure is still absent today. The NGO she was working for closed in 2010, and she subsequently secured her current employment. Her job consists primarily of promoting public health programs to the 300 families in the area she is responsible for (and in which she lives) and mediating residents’ access to public healthcare services. In 2014 Raimunda returned to school and enrolled in a private college to become a nurse. Raimunda is one of the 29 million Brazilians who were lifted out of extreme poverty by Workers’ Party (Partido dos Trabalhadores, PT) policies between 2003 and 2014 (Neri, 2014), and who benefited from social‐inclusion policies such as the quota programs for college.

Even though she feels that over the years changes in the functioning of the local health clinics made the work of health agents less impactful, because of cuts in social spending and Brazil's 2014 economic crisis, she is well received in all homes in her area. When I asked her if she had any reservations in doing her work, she replied: “In my area there is no such thing as being afraid of them. One thing that we managed to do in the past remains there. I am only afraid of being caught in the middle of a shootout.” By “one thing that we managed to do in the past” she refers to creating and maintaining the reputation of being *uma pessoa de bem* (a trustworthy person), a reputation she built over many years. Being close to the community's problems, as a health worker and a neighbor, and continuing her involvement in activist work around a progressive party that she supports, inspired Raimunda to make the improvement of her community a personal objective. How does she do that, besides doing her job?

Though dialogue*, orientando* (guiding). I am going into the house of a person who didn't manage to get a health exam and tell them: “Go to the justice! You have a right; the Constitution grants you this right!” Today, I do a lot of that.

She also tries to convince teenagers to go to school and to aspire to a better life as teachers or health workers like herself, because such jobs come with the right to a state pension and stability. Raimunda encourages them to study and to become lawyers or doctors, but the teenagers regard such jobs as reserved for the *filhos de rico* (wealthy children) who can afford to attend private schools. These insights echo Elizabeth Leeds's (2007) observation about the failure of public schools to function in violence‐prone areas in Rio de Janeiro, in the end reproducing existing problems by not serving as channels for local youth to access formal jobs. Raimunda also encourages girls to avoid pregnancy by using contraceptives and boys to use condoms. She proudly reports that in her community there are only a few pregnant teenagers. To Raimunda, the main challenge is to convince young children to avoid becoming part of the drug trade. By mentoring these youths and helping them avoid the *mundo do tráfico* she demonstrates a form of subversive care. These young people would be looked down upon in public spaces such as buses and downtown shopping centers. Many activists say that even certain school principals do not acknowledge the potential of these youths to transform their lives and attain their aspirations of a better life.

Raimunda regards violence as structural, and therefore maintains that reducing urban violence requires systemic changes to create a society where everyone can achieve their potential. Raimunda's understanding of violence cannot be separated from poverty and development and is in accord with Amartya Sen's theory of development as freedom (2000). She talks about the need for children to have *visão* (vision) and encourages them to imagine their lives differently than their parents’ and to truly believe that they can achieve their potential. She tells teenagers about her own story and the stories of other locals who go to college and get good jobs, thanks to the successful affirmative action programs implemented by PT (Penha Lopes, 2017). Besides doing the counseling and her formal job, she helps them the way she can:

A few months ago, a guy came with a bullet in his leg. I told him, “I am not God, I am not a doctor, I don't have enough resources to do what you want me to do.” He wanted me to remove the bullet, right? He told me he didn't have any options and that he takes all the responsibility. . . . “You will lose blood; you might get an infection.” In the end, I removed it with a nail file. The wound needed to be dressed. I was coming back from college, and he was waiting for me, you understand? It was 11:30 p.m., midnight. . . . In the end he got well. The doctor [from the health clinic] helped me in a way. I told him, “Doctor, I have this person, he got a bullet, he has an infection, can you help me with antibiotics?” . . . This is not part of my job, but when you live in a place like this, people know about your life. So, denying something like that is not good. It's bad for my living with them, but also for me as a human being because I know that he might die without seeking treatment. That's because this person is denied the right to health. If he catches a bullet, he cannot go to the health clinic; so he will go to the UPA [Unidade de Pronto Atendimento, or emergency room] and there they will call the police right way. So, from the hospital he goes straight to the prison. So, isn't his right to health denied?

Raimunda shares an understanding of health as a human right, an idea promoted by social movements that she has been part of in Fortaleza since the 1940s (Scott Jerome, 2014). I traced Raimunda's trajectory because her understanding of politics and activist practices is not unique. Conjunto Palmeiras has various people like her, and so do other poor neighborhoods in Fortaleza and elsewhere in Brazil (Fahlberg, 2018; Savell, 2015) and in Latin America (McIlwaine and Moser, 2007). In the next section, I present ethnographic examples to illustrate how such activists manifest their subversive care toward members of the drug gangs.

## THE SUBVERSIVE DIMENSION OF CARE

In 2017, different gangs delimited and controlled different parts of the neighborhood, locally called *territórios* (territories), and often residents were not allowed to cross the invisible boundaries of their *território* and access public resources or NGO services. In this section, I discuss how activists maintain relations of mutual trust with people labeled as “marginals” and therefore actively destigmatize the urban periphery.

### Trusting and subversively caring for the “marginals”

Trust is inherent in the practice of (subversive) care. The question of respect in relation to drug gangs has long been a topic of analysis in anthropology and other social sciences (Anderson, 1999; Bourgois, 2003; Vigil, 2003). In Elijah Anderson's ethnography of an American inner city, he defines respect as “an external entity, one that is hard‐won but easily lost—and so must constantly be guarded” (1999, 33). The code of the street emphasizes public displays of achieving respect, often using violence. Respect in the situations described here does not involve violence as a repertoire of action (Auyero et al., 2014) or confrontational politics. On the contrary, it involves respect for each other as citizens and residents of the same neighborhood, who do not only have to live in a symbiotic relationship (Koonings and Kruijt, 2007) but respect each other as humans, above all. Ethnographies about Rio de Janeiro have discussed similar findings about the relationship between drug gangs and local organizations, where such dynamics range from donations to peaceful coexistence (Leeds, 2007). Here I emphasize that it is not particular institutions that establish peaceful and respectful relations with the drug gangs, but individuals who have a history of civic work in the neighborhood and whose work is perceived by others as worthy of respect.

Essential to moving from one territory to another and conducting one's work is the underlying belief that “They know us, they know our history,” as intimated by Dona Maria, a social worker with one of the local NGOs:

Whenever I go to do visits, it's calm. I recently went to one of these areas, but people warned: “There might be shootings tonight.” I go everywhere and I am not afraid. When I get there, many people know me. I see teenagers that I took care of as kids. I have many students there. I have many acquaintances, so I am not afraid. I am only afraid of the *caras novas* (literally, new faces; here, new gang members) from outside who don't know our history.

In her youth, Dona Maria worked for the local Nutrition Center, taking care of newborns and toddlers suffering malnutrition. The NGO she currently works for has been active in the neighborhood for longer than any other organization, and employees like her who regularly visit project beneficiaries are well known by locals. Calculations related to movement in public spaces are tied to local histories of race, class, ethnicity, and gender relations, as indicated by anthropologists in Latin America (Collins, 2015; McCallum, 2005). What I want to emphasize here is that activists’ presence in areas avoided by other community members is made possible by the acknowledgment of their care work and therefore of their trustworthiness. Having cared for these youths as children and being embedded in social work in the neighborhood for many decades created bonds of mutual trust. These bonds notwithstanding, as Dona Maria mentioned here, the presence of new people in the drug trade threatens the local ties she built over the years.

Similarly, Marta, a former activist with the local liberation theology movement, currently active in the Catholic church in the city, emphasizes the centrality of respect in her relationship with teenagers who are members of drug gangs. She describes below how her respect stems from a critical understanding of urban violence:

Violence and drugs exist everywhere, but they seem more visible in places with poverty. For example, you see Conjunto Palmeiras as the most dangerous place because here people are poor. Drugs and violence are everywhere but this doesn't have to be something that threatens our struggles. I tell you, sometimes I leave home at 2 a.m. in the morning and go to Jardim Ceará, Bonsuceso [*territórios*]. We don't have any problems because we know each other. We have families living in these places. . . . We know them, and they know us. It's hard to see them killing *uma pessoa de bem* in Palmeiras because they respect us. . . . This problem with drugs is bigger than that because there are many white‐collar people who earn from this business. It's all about capital, and our job is to rescue *humanização* (humanization). . . . We must be engaged in this rescue.

By *pessoas de bem* Marta refers to residents who do not have any direct engagement with *tráfico* and are respected in the community, a respect they have earned through many years of living and working there. A *pessoa de bem* is also not someone who would betray them to the police or be involved with rival drug gangs. Marta also draws attention to a process of dehumanization, because, as many activists lament, “Life lost its meaning.” Not only do crimes take place easily—for a phone or small amounts of money—but suffering and death have become a spectacle that too many residents consume through the mass media. Humanizing the youths means treating them with respect and dignity, and therefore subverting narratives that label them “marginals,” therefore criminals, and unworthy of respect or care. Marta also critically assesses what violence actually means, when she says that those who profit from this business are the “white‐collar people,” not the youths who live at the margins of the city.

### Being in movement and “breaking the logic of fear”

A large part of understanding local problems stems from being, as many activists put it, *no movimento* (in movement). By *no movimento* activists do not mean a particular social movement, but the practice of visiting people's homes, listening to their stories, offering guidance, and often sharing their own social networks.

Beatriz, the woman introduced at the beginning of the article, draws on her experiences during her formative years with the liberation theology movement in acknowledging the struggles of those who are most vulnerable and yet often ignored by NGOs or other institutions. Like other community activists, she regards gang members’ participation in the drug economy as episodic and temporary, not as a definitive moral trait, as others do when they label gang members as *marginais*.

As indicated, activists regard violence as being structural. They accuse the media of creating and perpetuating images of their neighborhood as being “poor, therefore violent.” Erika Robb Larkins's (2015) ethnography of one of Rio de Janeiro's favelas discusses how the favela‐as‐a‐dangerous‐zone represents a spectacle largely constituted through the mass media. Robb Larkins argues that “the prevalence of violent spectacle and this circulation in the media contribute to the formation of the ‘hyperfavela,’ where the favela as it is, constituted through the media, is read as reality” (14).

The acknowledgment that the “hyperfavela” is not the “real” favela is made clear by Eliane, a local activist also involved with the women's movement and the Black movement:

This industry of fear interferes with people's mobility. “I am not going to college because I am scared; I am not going to the protest because there can be a misunderstanding; and the bandit appears and kills everybody.” This prevents people from organizing. They [the people] say, “Yes, it's bad but I am too scared to take the risk.” . . . Even though *fico com medo* [it scares me too], I look left, I look right, but I don't let this stop me. Because if I am always thinking that I am not going to this or that area, I will end up staying at home; all areas where I go are dominated by *tráfico*. I have to think that I am here for the community. . . . I am breaking this logic of fear!

Here Eliane does not imply that people stopped their lives when gangs came into the neighborhood, but she criticizes those who ceased to engage in collective action to solve local problems. Her activism includes challenging the media narrative that perpetuates fear and immobilizes residents, leading to inaction. She does not suggest challenging the new order imposed by the gangs but uses the legitimacy and trust built over the years to challenge normative assumptions about their neighborhood and remain engaged in vulnerable areas. The Pink Tide era shaped the political subjectivities of the urban poor in many ways (Junge, 2018), and my findings echo Charles Klein's (2019) research on activist groups in São Paulo's Zona documenting how previously poor citizens engage in progressive activism in their home communities. Eliane is one such activist who, like those discussed by Klein in São Paulo, benefited from social‐inclusion policies implemented from 2003 in Brazil, attended college, is now on the path to a PhD degree, and does not consider leaving her neighborhood but is engaged in a continued effort to improve it.

## CONCLUSION

In this article I introduced the term *subversive care* to discuss activist practices of women community leaders in Northeast Brazil. Such activist practices are gendered and politicized but also subversive—they seek to destabilize entrenched binaries such as center–periphery, associating the margins of the city with poverty and violence. Such geographical distinctions in Brazil are never only spatial but contain moral inferences about low‐income Brazilians. I located subversive care in literature about Latin American women's agency and explained why terms such as *resistance* or *militancy* cannot encapsulate the nature of this activist work. I then provided a general overview of Fortaleza, with a focus on the implications of drug‐related violence for the city. The rest of the article was dedicated to discussing ethnographic material that illustrates subversive care.

Many of the women engaging in subversive care presented here were at some point part of the liberation theology movement and are inspired by Christian values in their political thinking. These women are currently part of evangelical churches, religions of African origins, or do not attend any religious meetings at all. Instead of tying subversive care to a particular religious tradition, I regard it as culturally and historically embedded. In the case presented here, subversive care is an extension of insurgent citizenship practices in the twentieth century (Holston, 2008). The concept of subversive care can be used to describe the work of other activists elsewhere, for example, the work of activists who help migrants and refugees in border regions, especially when refugees are presented by the media or public officials as terrorists or criminals. Also, subversive care includes the provision of care to people from religious groups or with identities considered “repugnant.” It remains to be explored how these activist practices are embedded into local cultures and histories of “doing good.”

Visiting Conjunto Palmeiras in 2022, I noticed that many women whose stories I presented in this article started new civic work during the pandemic. One has mapped the *terreirors* (African religious groups) in Conjunto Palmeiras and is now in the process of raising visibility for members of the Candomblé and Umbanda religions. Another one started a project of providing food every month to a vulnerable part of the neighborhood, also serving the children and families of the gang leaders. Overall, activist work diversified and flourished in the neighborhood. Yet these efforts seem too isolated and disparate to unite their forces into a single movement that makes clear political demands. Moreover, in 2022 members of the evangelical churches seem to have even cut contacts with more progressive activists. It is yet to be seen how the next four years will potentially reunite not only Brazil but communities such as Conjunto Palmeiras and strengthen instead of weaken activist movements in the face of new global challenges.

## ENDNOTES

1 The term *popular educator* is inspired by Paulo Freire's work (1973) to describe education as liberation through consciousness raising (*conscientização* in Portuguese) or a process that develops critical thinking and the understanding of the world with its tensions and contradictions. According to Freire, this practice of learning and teaching popular education is imbued with a political transformative potential.

2 In this article I do not use pseudonyms when referencing places, but I use them when referring to research participants to protect their anonymity.

3 The Brazilian Institute of Geography and Statistics distinguishes five racial categories: white, Black, Indigenous, yellow/Asian, and *pardo* or Brown. According to the 2010 Brazilian Census, in Fortaleza 57.21 percent of the population identified as *pardo*, 36.28 percent as white, 4.91 percent as Black, and less than 2 percent as Indigenous or yellow (IBGE, 2023).

4 For more discussion on the increase in drug‐related violence in Fortaleza, its manifestations, and its impact, see the work of Luiz Fábio Paiva (2019) and the Laboratory for the Study of Violence at the Federal University of Ceará (https://lev.ufc.br).
